# Inactivation of SARS-CoV-2 on Surfaces by Cold-Plasma-Generated Reactive Species

**DOI:** 10.3390/bioengineering10030280

**Published:** 2023-02-21

**Authors:** Som V. Thomas, Krista Dienger-Stambaugh, Michael Jordan, Yuxin Wang, Jason Hammonds, Paul Spearman, Donglu Shi

**Affiliations:** 1 Department of Biomedical Engineering, College of Engineering and Applied Science, University of Cincinnati, Cincinnati, OH 45221, USA; 2 Division of Infectious Diseases, Cincinnati Children’s Hospital Medical Center and University of Cincinnati, Cincinnati, OH 45229, USA; 3 The Materials Science and Engineering Program, Dept. of Mechanical and Materials Engineering, College of Engineering and Applied Science, University of Cincinnati, Cincinnati, OH 45221, USA

**Keywords:** cold atmospheric plasma, COVID-19, virus inactivation, oxygen reactive species

## Abstract

A Cold Atmospheric Plasma (CAP) apparatus was designed and developed for SARS-CoV-2 killing as evaluated by pseudotyped viral infectivity assays. The reactive species generated by the plasma system was fully characterized by using Optical Emission Spectroscopy (OES) measurement under given conditions such as plasma power, flow rate, and treatment time. A variety of reactive oxygen species (ROS) and reactive nitrogen species (RNS) were identified from plasma plume with energies of 15–72 eV in the frequency range between 500–1000 nm. Systematic virus killing experiments were carried out, and the efficacy of CAP treatment in reducing SARS-CoV-2 viral infectivity was significant following treatment for 8 s, with further enhancement of killing upon longer exposures of 15–120 s. We correlated killing efficacy with the reactive species in terms of type, intensity, energy, and frequency. These experimental results demonstrate effective cold plasma virus killing via ROS and RNS under ambient conditions.

## 1. Introduction

Plasma, one of the four fundamental states of matter, is composed of a combination of neutral atoms, atomic ions, electrons, molecular ions, and excited and ground state molecules [1,2,3]. It can be produced by exposing gas, such as air or argon, to a strong electromagnetic field, which causes negatively charged electrons to dissociate from the nucleus. The charged particles in plasma possess substantial internal energies that can be utilized for altering surfaces. Currently, plasma treatment is utilized in surface and interface engineering to enhance adhesion, hydrophobicity, hydrophilicity, printability, corrosion resistance, selectivity, and for surface etching or cleaning [4,5,6,7,8,9,10,11]. The primary principle behind the plasma technique is that ionized and excited molecules generated by an electrical field directly impact the surface of the substrate. These activated molecules can modify the surface properties of substrates by etching, sputtering, or depositing on them. The plasma technique is an environmentally friendly process performed at room temperature. Therefore, it can be applied for surface modification and thin film deposition on a wide range of substrates such as metals, ceramics, polymer films, paper, glass, and nanoparticles [4,5,6,7,8,9,10,11].

Cold plasma or non-equilibrium plasma is a plasma state in which the electrons and heavier species (ions and neutrals) are not in thermodynamic equilibrium and the temperature of these heavy species (ions and neutrals) is lower than electron temperature [12,13], which is expressed in terms of the energy unit electron volt (eV). Cold atmospheric plasma (CAP) is a powerful tool that can generate ROS, RNS, charged particles, photons, and electric fields for biomedical applications via two mechanisms: “plasma killing” and “plasma healing”. The former is utilized in bacterial sterilization [14], treatment for fungal infection [15], degradation of both organic/inorganic contaminants in water treatment [16], virus inactivation [17], and apoptosis of cancer cells [18], while the latter can be used to enhance cellular oxidation in wound healing and modify surfaces for biological implants (hydrophobic/hydrophilic surface properties) [19]. These in vitro ROS and RNS are also responsible for promoting cell differentiation and apoptosis, as well as other biomolecular activities.

In the realm of biomedical applications, cold atmospheric plasma (CAP) has been employed to combat the COVID-19 pandemic by eliminating the virus [20,21,22,23,24,25]. The transmission of COVID-19 occurs through respiratory droplets originating from infected individuals’ coughs and sneezes. These droplets can contaminate various surfaces such as bedding, floors, walls, and objects, which uninfected individuals may meet. While the Centers for Disease Control and Prevention (CDC) have recommended measures such as the use of hand sanitizers, alcohol, and diluted sodium hypochlorite solutions to prevent such transmission, there is a lack of systematic studies to confirm their effectiveness in killing COVID-19. Additionally, it remains unclear how to effectively control the spread of COVID-19 through disinfection methods for everyday activities. Most disinfectants available are in liquid or gel forms, which are effective on hard surfaces or skin, but not on soft surfaces such as clothing, mail, printed materials, and human hair, which frequently encounter public surfaces and can serve as potential sources of COVID-19 spread if not promptly and adequately disinfected. Thus, it is crucial to develop a “dry” treatment method, such as cold atmospheric plasma, that can be easily and frequently applied to these everyday items.

In this study, we carried out SARS-CoV-2 killing assays using pseudotyped viruses via cold-plasma-generated reactive species in terms of their distributions and time dependencies that could be correlated to different in vitro biological processes. The goal of this study was also to investigate the efficacy of plasma treatment on SARS-CoV-2 pseudotyped viruses by controlling the parameters of the CAP device. We designed and developed a low-pressure plasma system for studies on the efficacy of plasma disinfectant.

## 2. Experimental Methods

### 2.1. Plasma Apparatus Design

**Figure 1:**
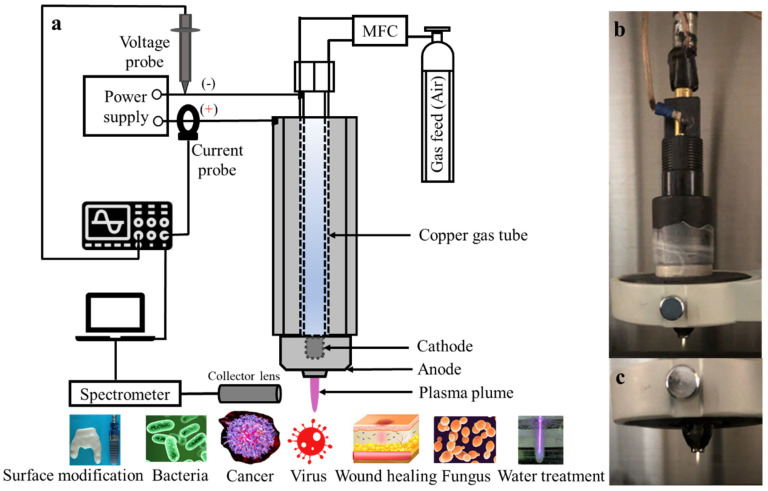
Schematic of diagrams: (**a**) the plasma apparatus design, (**b**) the P80 plasma cutting machine straight-handle torch, and (**c**) the plasma plume produced at room temperature under ambient pressure.

The schematic diagram of the plasma system is shown in Figure 1a. We utilized a commercial P80 plasma cutting machine straight-handle torch (Figure 1b) with a nozzle diameter of 1.5 mm to produce the cold plasma plume. As shown in Figure 1a, a DC power source (CX-600A, Chengdu Chuangyu Xinjie Technology Co., Ltd., Sichuan, China) was used to drive the CAP torch (Figure 1c), which could produce variable voltage between 5–60 kV, 400 W output. The CAP torch electrode tip was connected as the cathode and the tip nozzle acted as the anode. The applied voltage was measured by using a high voltage probe (Precision USB 100 kV high voltage Probe, CPS HIGH VOLTAGE, Tigard, OR, USA) and the current by using a current monitor (CM-100-S, ION PHYSICS CORPORATION, Fremont, NH, USA); both were connected to a digital oscilloscope (SDS1204X-E—200 MHz/4 Channel, SIGLENT TECHNOLOGIES CO., LTD, Shenzhen, China). The electrical discharge voltage and current were found to be 9.64 kV and 40 mA, respectively.

In the plasma virus killing experiments, the flow rate of the feed gas was controlled using a SmartTrak^®^100 Digital Mass Flow Controller (Sierra Instruments, Monterey, CA, USA). We applied a high voltage at ~40 kV, current ~8 mA, and fed air (purity: 99.98%) through the plasma nozzle with a flow rate of 7 standard liters per minute (SLPM) to produce a plasma plume under ambient atmosphere (Figure 1c). As shown in Figure 1c, the plasma plume generated was uniform, with a length of about 15 mm and diameter of 5 mm. It could reach the sample well bottom uniformly for effective virus killing. Optical emission spectroscopy (OES) was performed using a photo spectrometer AvaSpec, ULS4096CL-EVO-UA-10 (Avantes, Apeldoorn, The Netherlands) connected to an optical fiber to analyze and identify the reactive species (RS) produced by the CAP torch between wavelengths of 200–1100 nm. Plasus Specline−AMS (Ver 2.1, PLASUS GmbH, Mering, Germany) Plasma characterization software was used to identify individual species with various characteristics such as frequency, energy, and intensity.

**Figure 2:**
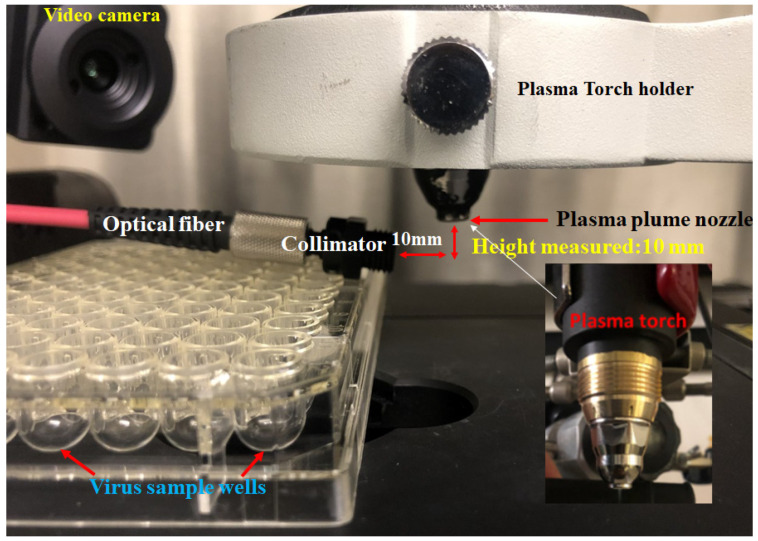
Photograph showing the experimental setup for the optical emission spectroscopy (OES) measurement.

The optical emission spectroscopy (OES) measurement was performed by horizontally positioning the optical fiber sensor radially 10 mm away from the tip of the plasma plume nozzle and 10 mm below the nozzle tip (see horizontal red arrow in Figure 2). The height of the optical sensor was varied within 10 mm vertically below the plasma nozzle, as indicated by the red arrow in Figure 2, to determine the plasma strength profile. The purpose of the OES experiment was to identify the types of prevalent RS at different time intervals and air flow rates.

### 2.2. Virus Construction and Infectivity Experimental Methods

Cell lines: The human embryonic kidney epithelial cell line 293T was obtained from ATCC and cultured in Dulbecco’s modified Eagle medium (DMEM) with 10% heat-inactivated fetal bovine serum (FBS), 1% l-glutamine (Gibco, Waltham, MA, USA), and 100 U/mL penicillin–streptomycin (Gibco). Human-angiotensin-converting enzyme 2 (hACE2) was amplified via PCR and cloned into the Xho1 and Xba1 sites of pLVX-Puro (Clontech, Mountain View, CA, USA), confirmed by DNA sequencing. Lentivirus generated using a third-generation system comprised of pLVX-hACE2-Puro, psPAX2, and pMD2.G (Addgene plasmid # 12260; http://n2t.net/addgene:12260 (accessed on 13 December 2022); RRID:Addgene_12260 and Addgene plasmid # 12259; http://n2t.net/addgene:12259 (accessed on 13 December 2022); RRID:Addgene_12259), gifts from Didier Trono) was utilized to produce a 293T cell line that stably expressed hACE2 under puromycin selection (1.5 µg/mL, Invivogen). Stable expression was confirmed by flow cytometric analysis using hACE2-specific antibodies. Cells were expanded and cryopreserved for use in viral infectivity assays.

### 2.3. SARS-CoV-2 Pseudotyped Lentivirus Production

Lentivirus was produced in 293T cells utilizing a third-generation three-plasmid system. Single-round lentiviruses were produced by using JetPrime (Polyplus) transfection reagent to co-transfect pTMLW, psPAX2, and a pseudotype envelope expression plasmid, either SARS-CoV-2 (Wuhan strain) spike, pCMV SARS-CoV-2 Spike CΔ19 R682Q [26], or the positive control vesicular stomatitis virus envelope (VSV-G) pMD2.G.

The pTMLW transfer vector contained a 3′ and truncated 5′ long terminal repeat, expressed a firefly luciferase reporter transgene under the control of an RSV promoter, and was a gift from Punam Malik at Cincinnati Children’s Hospital Medical Center. The psPAX2 packaging plasmid ensured replication-incompetent lentivirus production. After 48 h had passed post-transfection, the virus-containing cell culture supernatant was harvested, pooled, and clarified via centrifugation at 2500× *g* for 10 min at 4 °C. Supernatants were filtered through a 0.45 μm Nalgene filter before being aliquoted, snap-frozen, and stored at −80 °C.

### 2.4. SARS-CoV-2 Pseudotyped Lentivirus Infectivity Assay

To measure infectivity, hACE2 293T cells were seeded in a 96-well plate and viral supernatants were applied with 5-fold serial dilutions in the presence of 8 μg/mL DEAE-Dextran. We assayed cells for luciferase reporter gene expression at 48 h post-infection by removing 100 μL of culture media and replacing it with 100 μL Britelite substrate (Perkin Elmer, Waltham, MA, USA). The cells, remaining media, and Britelite were mixed until homogenous, then 150 μL of lysate mix was transferred to a black 96-well plate for measurement of emitted light. Luminescence was measured within 10 min using a Bio-Tek Synergy Neo2 luminometer and is reported as the mean RLU of four replicate wells +/− SD. TCID50 values were calculated for quadruplicate samples using Reed–Muench methods. RLUs from uninfected cells were averaged to determine the assay background. Infection positivity was defined as an RLU greater than 2.5 × background RLU [27,28,29].

### 2.5. Preparation of SARS-CoV-2 Pseudotyped Lentivirus for CAP Treatment

Viral supernatants (100 μL) of known titer were applied to a standard 96-well round-bottom cell culture plate (Corning 351177, Tewksbury, MA, USA). The plate was placed inside of a desiccated chamber within a laminar flow hood and virus supernatant was dried overnight under ambient sterile conditions. The desiccated virus was then subjected to the cold plasma plume for varying time intervals from 0 (untreated) to 120 s. The virus was also treated with non-energized air at 7 SLPM for 120 s to control for any effects that may be due to air turbulence alone. The treated virus was then reconstituted in 100 μL of cell culture medium and tested at various dilutions for infectivity in the SARS-CoV-2 pseudotyped lentivirus infectivity assay.

## 3. Results

### 3.1. Spectroscopy Characterization of Cold Plasma-Generated Reactive Species

**Figure 3:**
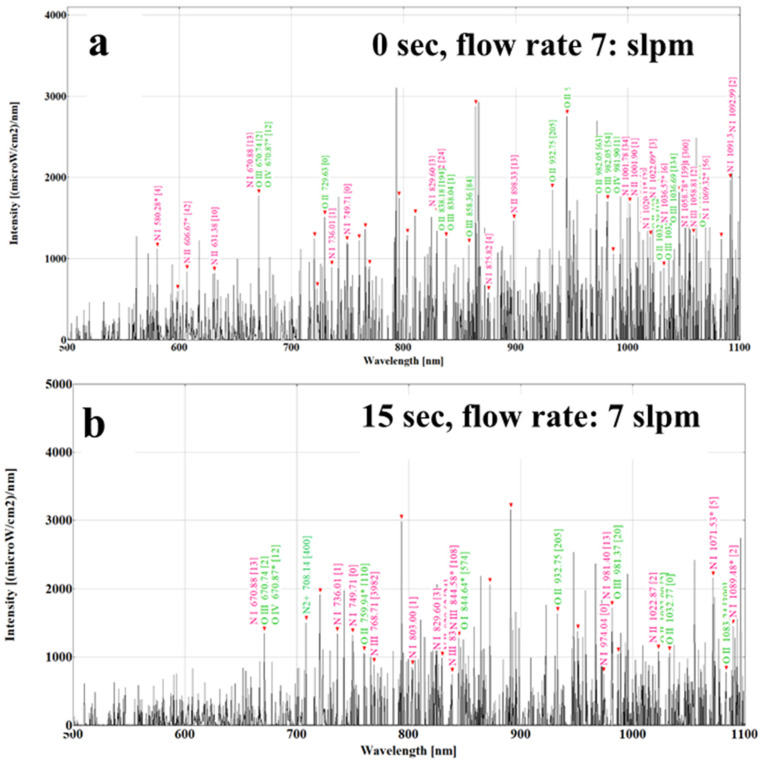
Intensity vs. wavelength for oxygen and nitrogen species generated by plasma at (**a**) 0 s and (**b**) 15 s with flow rate 7 SLPM.

We carried out OES experiments at a flow rate of 7 SLPM, with spectroscopic analysis at 0 s and 15 s. Figure 3 shows various plasma reactive species produced at 0 s (Figure 3a) and 15 s (Figure 3b). Reactive oxygen and nitrogen species (RONS) include two classes of chemically reactive molecules containing oxygen (reactive oxygen species, ROS) and nitrogen (reactive nitrogen species, RNS). Both classes are referred to as RONS. The majority of RONS carries unpaired electrons, also known as free radicals. As can be seen in this figure, both ROS and RNS were generated at different wavelengths and intensities. Numerous RONS were observed at both 0 s and 15 s, indicating their prevalent and frequent appearance under plasma excitation.

Table 1 and Table 2 summarize the parameters of RONS at 0 s and 15 s, respectively, including wavelengths, intensity, ionic species excited state (single/multiple), identified lines, relative intensity, energy (lower/upper), transition state (lower/upper), and quantum number (lower/upper). In these tables, N I, N II, N III and O I, O II, OIII, OIV debnote RNS and ROS, respectively.

As shown in Table 1 and Table 2, there are numerous ROS and RNS at 0 and 15 s with different characteristics. While the energies of ROS are generally higher (~29–72 eV), those of RNS tend to be smaller (~12–43 eV). From Figure 3, Table 1 and Table 2, one can see significant RONS being generated by plasma, which could be responsible for virus killing or deactivation.

**Figure 4:**
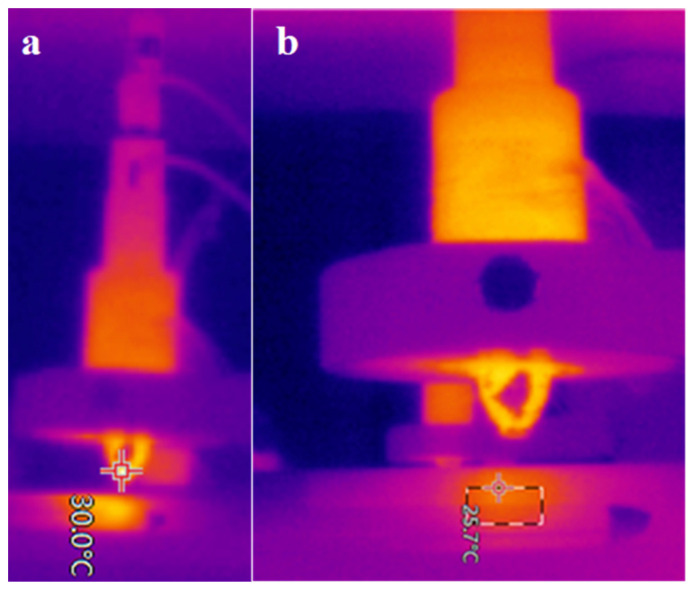
Thermal images of CAP torch and microwell plate. (**a**) The maximum temperature at the nozzle is 30 °C. (**b**) Assay well area measured inside the rectangle is 25.7 °C.

To verify that the plasma treatment was performed at ambient temperature, the temperature profiles of the experimental set up were monitored using a thermal camera (Benchtop PCB Thermal Imaging Camera, Teslong 260 × 200 Infrared Thermal Analyzer, Irvine, CA, USA). Figure 4 shows the thermal images of the CAP torch (Figure 4a) and microwell assay plate where the viruses are contained (Figure 4b). As shown in Figure 4a, the temperature of the plasma nozzle was 30 °C, while that at the assay well area was 25.7 °C (Figure 4b). These thermal images indicate that the plasma was at room temperature, which is characteristic of CAP.

### 3.2. SARS-CoV-2 Pseudotyped Viral Infectivity Assays

To develop a suitable model of surface contamination, we applied SARS-CoV-2 pseudotyped virus to a polystyrene surface and demonstrated that infectious virus was still present even after overnight desiccation under ambient conditions. We used a VSV-G-pseudotyped lentivirus as a positive control for the infectivity assays. We also controlled for the potential displacement of virus from the solid surface due to air pressure alone that was generated during CAP treatment. The infectivity assay performed as expected, based on the ability of VSV-G-pseudotyped virus to infect cells at a high TCID_50_. We did not observe any decrease in viral infectivity after treatment of viruses with air alone, as compared to virus that was only desiccated (data not shown).

**Figure 5:**
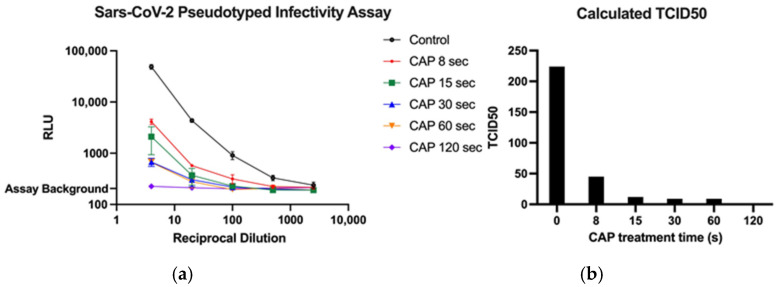
(**a**) RLU vs. reciprocal dilution for the SARS-CoV-2 pseudovirus infectivity assay, (**b**) TCID_50_ vs. CAP treatment time.

Based on the plasma setup (Figure 1) and RONS characterization (Figure 3, Table 1 and Table 2), we conducted cold plasma treatment of SARS-CoV2 spike pseudotyped virus. We confirmed that the cold plasma plume was not above ambient temperature, and the plume length was sufficient to reach the bottom of the assay well containing desiccated virus. Figure 5a shows the infectivity assay results measured as luciferase counts (RLU) at various dilutions of the air-treated control viral inoculum as well as the inoculum after treatment with CAP for the indicated times. Measurement of luciferase activity over multiple dilutions allowed for calculation of the TCID_50_. Figure 5b shows the calculated TCID50 for each condition measured in Figure 5a, revealing a substantial reduction in remaining infectious units following treatment at 8 s and further reduction upon longer treatment.

**Figure 6:**
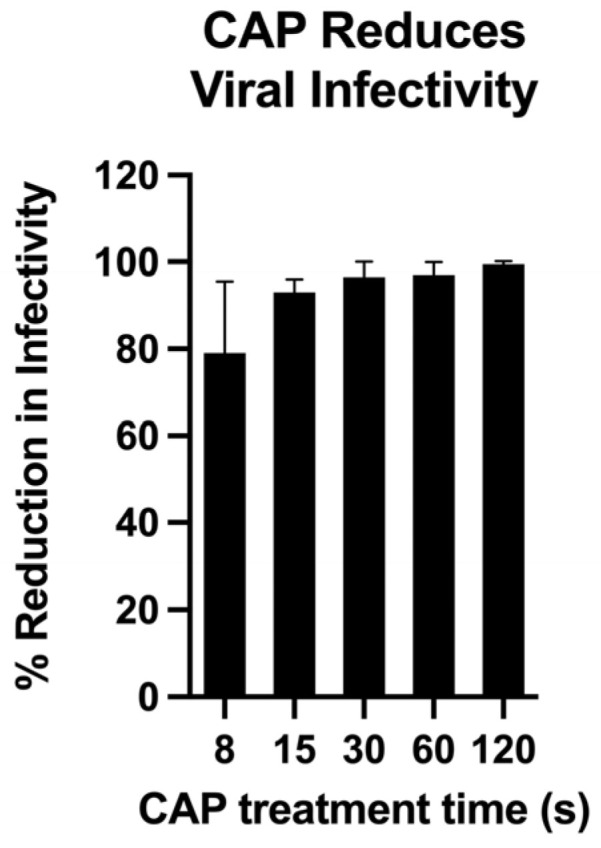
% Reduction in infectivity vs. CAP treatment time.

To illustrate the reproducibility and efficiency of killing, the data from Figure 5b are presented as percentage reduction in infectivity in Figure 6. Cold plasma treatment resulted in a mean reduction in infectivity of 79% in as little as 8 s that increased to 93% by 15 s (*n* = 2). Further reduction occurred at 30 and 60 s, with a sustained mean reduction of 97% infectivity, and a near return to background RLU with 99% reduction at 120 s (*n* = 5). As is evident from this figure, even very short times of application of the plasma plume to the surface resulted in a significant reduction in infectivity.

## 4. Discussion

Cold atmospheric plasma (CAP) has been investigated for its potential to inactivate SARS-CoV-2, the virus responsible for COVID-19 [30,31,32]. Studies have shown that CAP can effectively inactivate SARS-CoV-2 on surfaces and in the air. The mechanism by which CAP inactivates the virus is not yet fully understood, but it is thought to involve the production of reactive oxygen and nitrogen species (RONS) that can damage the virus’s genetic material, rendering it unable to infect cells. Prior research indicates that RONS can cause harm to various cellular organelles and biological processes [33,34,35]. Nevertheless, RONS can also serve as a signaling molecule within host cells, prompting the activation of immune responses to combat viral infections. For instance, RONS produced by the host cells can stimulate the inflammasome, a protein complex that prompts the production of cytokines. These signaling molecules activate immune cells to counter the virus. On the other hand, RONS can impede viral infections by directly impairing the virus’s genetic material, reducing its infectiousness. That being said, RONS can result in oxidative stress within host cells, causing cellular damage, inflammation, and apoptosis [36,37,38].

As shown in Table 1 and Table 2, under different plasma conditions, RONS were directly generated in the plasma discharge zone. Both reactive oxygen species (ROS) and reactive nitrogen species (RNS) are excited/neutral molecules containing a minimum of one oxygen atom and single/multiple unpaired electrons. These ensembles consist of free radicals such as superoxide anion, hydroxyl, hydroperoxyl, singlet/doublet/triplet/quartet oxygen, free nitrogen, and free hydrogen species in some cases. The production of a surplus of free species adds to oxidative stress on the cell surface, inducing molecular- and cellular-level impairment of structures. These in vitro ROS and RNS become signaling molecules when impinging on the cellular surface, leading to chemical imbalances and disruptive effects to proteins (aggregation, denaturation), lipids (peroxidation), carbohydrates, and nucleotides (changes in the DNA structure) of the cellular species. As also shown in Table 1 and Table 2, the intensities of RONS are significantly high, with a maximum on the order of ~70 eV for ROS and ~40 eV for RNS responsible for significant virus killing efficacies within a short period of time.

Infection of ACE2 expressing cells by SARS-CoV-2 relies on the trimeric SARS-CoV-2 spike glycoprotein. We produced a lentivirus bearing a structurally and functionally similar spike glycoprotein as a surrogate for the SARS-CoV-2 virus itself, which requires BSL3 conditions for cultivation and quantitation. Pseudotyped viruses were easily recoverable from a dry surface, providing a readily quantified measure of infectivity from untreated or CAP-treated wells. Our results demonstrate the ability of CAP to diminish viral infectivity in a time-dependent manner in this sensitive and quantitative assay. The efficacy of CAP treatment on SARS-CoV-2 viral infectivity was highly reproducible. Cold plasma treatment for as little as 8 s produced significant killing, while killing of residual infectious virus was further enhanced upon longer exposures. Application of the treatment for 120 s effectively reduced infectivity measurement to background levels (100% killing). We recognize that the shorter times of application (8–15 s) will be more practical for future applications of the technology as a mode of reducing virus transmission from surfaces.

## 5. Conclusions

We found numerous reactive oxygen and nitrogen species (RONS) in a wide spectrum of frequencies with high energies that were the most likely to be responsible for virus killing. Experimental results from this study provide proof of concept for the ability of cold plasma to inactivate SARS-CoV-2 on surfaces. One key outcome of this study is the correlation between the virus killing efficacy and the reactive species that are generated by the plasma device. This plasma system was fully characterized by the reactive species generated under different conditions such as flow rate and time. The new methods to generate a stable and effective cold atmospheric plasma plume were shown to be effective, as evidenced by the results of killing SARS-CoV-2 pseudovirus. Future studies are needed to expand on the work described here, including application of cold plasma against other enveloped viruses on surfaces and potentially the identification of the particular RONS that are most effectively deployed for the inactivation of viruses.

## Figures and Tables

**Table 1:** Reactive species identified via OES at 7 slpm at 0 s, with 30 RNS and 18 ROS.

Wave-Length (nm)	Intensity [(µW/cm^2^)/nm]	Element *	Line Identified (nm)	I (Rel.)	Energy (eV) Lower–Upper	Transition Lower–Upper	Quantum Number Lower–Upper
580.241	1121.53	N I	580.2836	4	11.84–13.98	3p 4P–5d 4F	2½–2½
580.241	1121.53	N I	580.3043	3	12.13–14.26	3p ^2^P–8s ^2^P	1½–1½
631.294	820.914	N II	631.3809	10	25.65–27.62	4p ¹S–6s ^3^P°	0–1
670.831	1775.77	N I	670.8759	13	11.84–13.69	3p 4P°–4d 4D	1½–1½
670.831	1775.77	O III	670.736	2	47.03–48.87	4d ^3^D°–3s ^3^D	2–3
670.831	1775.77	O IV	670.866	2	71.54–73.39	3s 4P–4d 4D°	1½–½
670.831	1775.77	O IV	670.866	12	71.54–73.39	3s 4P–4d 4D°	1½–1½
729.647	1512.35	O II	729.6294	0	28.86–30.56	3d 4D–4p 4P°	3½–2½
735.934	895.199	N I	736.0126	1	12.01–13.69	3p ^2^D°–4d ^2^D	2½–1½
749.633	1197.4	N I	749.7122	0	13.24–14.90	4p 4D–3d’ ^2^G	2½–3½
829.625	1541.9	N I	829.6007	3	12.13–13.62	3p ^2^P–5s 4P	1½–1½
829.625	1541.9	N II	829.621	24	23.57–25.07	3d ¹P°–4p ¹P	1–1
838.084	1246.14	O II	838.177	194	30.47–31.95	4p 4D°–5s 4P	½–½
838.084	1246.14	O III	838.036	1	45.44–46.92	4p ^3^D–3p ^3^P°	1–1
858.346	1166.76	O III	858.361	84	54.37–55.82	4p’ 5D°–4d’ 5P	4–3
898.415	1465.49	N II	898.3277	13	26.03–27.41	4d ¹D°–5f ^2^[2½]	2–3
932.692	1845.99	O II	932.7453	205	31.63–32.96	4d 4P–5f 4D°	1½–½
945.743	2754.02	O II	945.789	34	28.51–29.82	3p ^2^D°–4s ^2^P	2½–1½
945.743	2754.02	O II	945.8429	17	28.51–29.82	3p’ ^2^D°–4s ^2^P	2½–1½
981.981	1702.36	O II	982.055	63	30.80–32.06	4p ^2^P°–5s ^2^P	½–1½
981.981	1702.36	O III	982.047	54	45.94–47.20	4p ^3^P–4d ^3^P°	2–2
981.981	1702.36	O IV	981.895	1	73.96–75.22	4f 4D–3p 4P°	1½–2½
1001.81	1674.33	N I	1001.783	34	11.76–12.99	3p 4D°–3d ^2^F	2½–2½
1001.81	1674.33	N II	1001.903	1	26.21–27.45	4f’ ^2^[4½]–5g’ ^2^[2½]°	4–3
1020.2	1266.48	N I	1020.112	1	13.68–14.90	4f ^2^D [3]–3d’ ^2^G	3½–4½
1020.2	1266.48	N I	1020.113	2	13.68–14.90	4f ^2^D[3]–3d’ ^2^G	2½–3½
1020.2	1266.48	N I	1020.122	0	13.68–14.90	4f ^2^D[3]–3d’ ^2^G	3½–3½
1020.2	1266.48	O II	1020.23	1	29.59–30.80	4s 4P–4p ^2^P°	½–½
1022.12	1643.94	N I	1022.089	3	13.20–14.41	4p ^2^S–10d 4P	½–½
1022.12	1643.94	N I	1022.089	1	13.20–14.41	4p ^2^S–10d 4P	½–1½
1032.25	884.052	O II	1032.284	1	30.75–31.95	4p ^2^D°–5s 4P	1½–½
1032.25	884.052	O III	1032.177	454	44.28–45.48	4s ^3^P°–4p ^3^D	2–3
1036.63	1342.98	N I	1036.569	6	11.84–13.03	3p 4P–3d ^2^D	½–1½
1036.63	1342.98	N I	1036.574	2	13.26–14.46	4p 4P–12d ^2^D	½–1½
1036.63	1342.98	O III	1036.687	134	44.24–45.44	4s ^3^P°–4p ^3^D	1–1
1051.37	1579.21	N I	1051.341	300	11.84–13.02	3p 4P°–3d 4D	½–½
1058.73	1288.58	N I	1058.655	4	13.25–14.42	4p 4D–12s 4P	3½–2½
1058.73	1288.58	N I	1058.784	39	13.29–14.46	4p ^2^D–12d ^2^D	1½–1½
1058.73	1288.58	N III	1058.81	2	46.72–47.89	4p 4D–4d 4P°	1½–2½
1058.73	1288.58	O II	1058.83	10	31.76–32.93	4f 4F°–5d ^2^F	4½–3½
1069.35	1383.91	N I	1069.278	1	13.24–14.40	4p 4D–10d 4D	2½–1½
1069.35	1383.91	N I	1069.278	4	13.24–14.40	4p 4D–10d 4D	2½–2½
1069.35	1383.91	N I	1069.278	2	13.24–14.40	4p 4D–10d 4D	2½–3½
1069.35	1383.91	N I	1069.317	56	11.84–13.00	3p 4P–3d ^2^F	2½–3½
1069.35	1383.91	N I	1069.424	0	13.24–14.40	4p 4D–11s ^2^P	½–1½
1069.35	1383.91	N I	1069.424	2	13.24–14.40	4p 4D–11s ^2^P	½–½
1091.32	1964.13	N I	1091.364	1	11.84–12.98	3p 4P–3d ^2^P	1½–½
1092.95	2458.16	N I	1092.991	2	13.20–14.34	4p ^2^S–8d 4D	½–½

*: N I, N II, N III and O I, O II, OIII, OIV are denoted RNS and ROS, respectively.

**Table 2:** Reactive oxygen species identified via OES at 7 slpm at 15 s, with 20 RNS and 15 ROS.

Wave-Length (nm)	Intensity [(µW/cm^2^)/nm]	Element *	Line Identified (nm)	I (Rel.)	Energy (eV) Lower-Upper	Transition Lower-Upper	Quantum Number Lower-Upper
670.831	1345.65	N I	670.8759	13	11.84–13.69	3p 4P°–4d 4D	1½–1½
670.831	1345.65	O III	670.736	2	47.03–48.87	4d ^3^D°–3s ^3^D	2–3
670.831	1345.65	O IV	670.866	2	71.54–73.39	3s 4P–4d 4D°	1½–½
670.831	1345.65	O IV	670.866	12	71.54–73.39	3s 4P–4d 4D°	1½–1½
735.934	1339.33	N I	736.0126	1	12.01–13.69	3p ^2^D°–4d ^2^D	2½–1½
749.633	1323.49	N I	749.7122	0	13.24–14.90	4p 4D–3d’ ^2^G	2½–3½
759.892	1057	O II	759.8285	15	28.86–30.49	3d 4D–4p 4D°	1½–2½
759.892	1057	O II	759.85	64	28.86–30.49	3d 4D–4p 4D°	1½–2½
759.892	1057	O II	759.9198	24	28.86–30.49	3d 4D–4p 4D°	2½–2½
759.892	1057	O II	759.9384	110	28.86–30.49	3d 4D–4p 4D°	2½–2½
759.892	1057	O II	759.9407	0	31.32–32.95	3d’ ^2^G–5f ^2^G°	4½–3½
768.715	888.608	N III	768.7057	3982	42.40–44.01	5d ^2^D–6f ^2^F°	2½–3½
803.055	851.363	N I	802.9967	1	12.12–13.67	3p ^2^P–4d ^2^P	½–1½
829.625	981.434	N I	829.6007	3	12.13–13.62	3p ^2^P–5s 4P	1½–1½
829.625	981.434	N II	829.621	24	23.57–25.07	3d ¹P°–4p ¹P	1–1
838.648	745.261	N III	838.639	85	36.86–38.33	3s ^2^P°–3p ^2^P	1½–1½
844.563	1276.25	N III	844.5766	4	42.54–44.01	5g ^2^G–6f ^2^F°	3½–3½
844.563	1276.25	N III	844.5766	108	42.54–44.01	5g ^2^G–6f ^2^F°	4½–3½
844.563	1276.25	O I	844.6247	115	9.52–10.99	3s ^3^S°–3p ^3^P	1–0
844.563	1276.25	O I	844.6359	574	9.52–10.99	3s ^3^S°–3p ^3^P	1–2
932.692	1637.01	O II	932.7453	205	31.63–32.96	4d 4P–5f 4D°	1½–½
973.976	747.988	N I	974.0385	0	10.33–11.60	3s 4P–3p ^2^S	1½–½
981.429	1722.65	N I	981.4021	13	11.76–13.02	3p 4D°–3d 4D	2½–3½
981.429	1722.65	O III	981.369	20	45.99–47.25	4p ¹D–4d ¹P°	2–1
1022.94	1080.7	N II	1022.867	2	26.21–27.42	4f’ ^2^[4½]–5g ^2^[3½]°	5–4
1022.94	1080.7	O II	1022.999	2	31.75–32.96	4f 4F°–5d ^2^D	1½–2½
1032.8	1059.48	O II	1032.772	0	31.76–32.96	4fF ^2^[4]°–5g ^2^[5]	4½–5½
1071.52	2159.25	N I	1071.526	5	13.27–14.43	4p 4P–11d 4D	2½–3½
1071.52	2159.25	N I	1071.526	0	13.27–14.43	4p 4P–11d 4D	2½–1½
1071.52	2159.25	N I	1071.526	1	13.27–14.43	4p 4P–11d 4D	2½–2½
1083.19	776.701	O II	1083.244	399	32.94–34.09	5f 4G°–4d’ ^2^D	3½–2½
1089.43	1450.25	N I	1089.399	1	13.24–14.38	4p 4D–10s 4P	1½–2½
1089.43	1450.25	N I	1089.437	2	13.24–14.38	4p 4D–9d ^2^D	2½–2½
1089.43	1450.25	N I	1089.476	2	13.24–14.38	4p ^2^D–11d ^2^F	1½–2½

*: N I, N II, N III and O I, O II, OIII, OIV denote RNS and ROS, respectively.

## Data Availability

Data are available in a publicly accessible repository.
